# Difficult Life Events, Selective Migration and Spatial Inequalities in Mental Health in the UK

**DOI:** 10.1371/journal.pone.0126567

**Published:** 2015-05-27

**Authors:** Helena Tunstall, Niamh K. Shortt, Jamie R. Pearce, Richard J. Mitchell

**Affiliations:** 1 Centre for Research on Environment, Society and Health, Institute of Geography, University of Edinburgh, Edinburgh, United Kingdom; 2 Centre for Research on Environment, Society and Health, Institute of Health and Wellbeing, University of Glasgow, Glasgow, United Kingdom; Institute of Psychiatry, UNITED KINGDOM

## Abstract

**Objective:**

Research has indicated that people moving towards neighbourhoods with disadvantaged socio-economic status have poor health, in particular mental health, but the reasons for this are unclear. This study aims to assess why people moving towards more socio-economically deprived areas have poor mental health. It focuses upon the role of difficult life events that may both trigger moves and damage mental health. This study investigates how mental health and socio-spatial patterns of mobility vary between people moving following difficult life events and for other reasons.

**Methods:**

Longitudinal analysis of British Household Panel Survey data describing adults’ moves between annual survey waves, pooled over ten years, 1996-2006 (N=122,892 observations). Respondents were defined as ‘difficult life event movers’ if they had experienced relationship breakdown, housing eviction/repossession, or job loss between waves. Respondents were categorised as moving to more or less deprived quintiles using their Census Area Statistic residential ward Carstairs score. Mental health was indicated by self-reported mental health problems. Binary logistic regression models of weighted data were adjusted for age, sex, education and social class.

**Results:**

The migration rate over one year was 8.5%; 14.1% of movers had experienced a difficult life event during this time period. Adjusted regression model odds of mental health problems among difficult life event movers were 1.67 (95% CI 1.35-2.07) relative to other movers. Odds of difficult life events movers, compared to other movers, moving to a less deprived area, relative to an area with a similar level of deprivation, were 0.70 (95% CI 0.58-0.84). Odds of mental health problems among difficult life event movers relocating to more deprived areas were highly elevated at 2.40 (95% CI 1.63-3.53), relative to stayers.

**Conclusion:**

Difficult life events may influence health selective patterns of migration and socio-spatial trajectories, reducing moves to less deprived neighbourhoods among people with mental illness.

## Introduction

Analysis of the health characteristics of migrants within developed countries has suggested that people moving towards socio-economically deprived neighbourhoods have relatively poor health [1–7]. The selective nature of migration means that residential mobility can be one of the processes that help to establish and maintain inequalities in health between areas with different levels of socio-economic disadvantage [8]. Research into the relationship between selective migration and spatial inequalities in health has however commonly treated migration processes as a ‘black box’. As a consequence the reasons why migrants have distinctive health and how their health status is linked to their propensity to move to more or less socio-economically deprived places are not fully understood [9].

Research that has considered the distinctive health characteristics of movers has primarily centred upon social and health selection. This suggests that the relatively good health of young adults movers [1, 4, 10, 11] reflects their socio-economic status [4], while poor health is found among movers in mid-life and older ages [12–14] in part because declines in health can trigger moves [14, 15].

Another possible factor shaping the health status of movers that has been less explored is ‘difficult’ life events. Some events could both trigger moves and have impacts upon health status. Relationship dissolution greatly increases propensity to move [16] and among marital and unmarried couples has direct impacts on health [17], with strong influences on mental health in the short term [18–20] and physical health in the longer term [21]. Unemployment also increases the probability of mobility [22] and damages health [23] with a particularly strong relationship with mental well-being [24, 25]. Housing arrears are associated with large negative psychological impacts that are equivalent to that of unemployment or marital dissolution [26] and repossession increases risk of mental distress [27].

Some research has considered how reasons for moves are associated with mover’s health. One UK study of pregnant women and mothers with infants found women relocating for negative reasons, including relationship breakdown, housing eviction or repossession, and problems with parents and neighbours, were more likely than other movers to have poor self-rated health and depression [28]. Some types of difficult life events may also prompt distinctive socio-spatial patterns of mobility. For example, unemployment is associated with greater likelihood of moves to more socio-economically deprived neighbourhoods [16].

There is therefore evidence indicating that difficult life events can harm health, trigger moves and increase the probability that a move will be to a more deprived area. This suggests that it is plausible that difficult life events could be one factor affecting selective patterns of migration between areas with different levels of deprivation. The aim of this study is to begin to assess this assertion by describing the relationship between difficult life events, mental health, residential moves and neighbourhood socio-economic disadvantage.

The study examines mental health because this aspect of health is strongly and proximately associated with difficult life events [18–20, 24, 25, 27]. It has also been demonstrated that people moving towards deprived neighbourhoods have highly elevated risk of mental illness [5–7]. Recent analysis of the British Household Panel Study (BHPS) that has assessed general health and self-reported mental health problems, found that poor mental health were particularly elevated among people moving towards more socio-economically deprived areas [7]. This study aims therefore to build upon these findings by assessing *why* people moving towards more socio-economically deprived areas have poor mental health, focussing upon one possible explanation, the role of difficult life events.

This study uses data from the BHPS to analyse the relationship between adults’ mental health, difficult life events and moves to more and less socio-economically deprived neighbourhoods in the UK. The study considers three difficult types of life events, closely related to mental health [18, 19, 24, 25, 27]: relationship breakdown, housing eviction or repossession and job loss.

The analysis tests a series of hypotheses about how difficult life events, mental health, residential mobility and neighbourhood deprivation are related. Firstly, people whose moves are associated with difficult life events have worse mental health than other movers. Secondly, people whose moves are related to difficult life events are, compared to other movers, more likely to move to deprived areas and less likely to move to less deprived areas. Thirdly, the poor health of people whose moves are related to difficult life events is more elevated among those moving to more deprived areas than those moving to less deprived areas. Lastly, poor mental health among movers that have experienced difficult life events is sustained following the move and therefore may have longer term implications for health geography.

## Methods

### British Household Panel Survey (BHPS)

This analysis uses data from BHPS an annual social survey completed 1991–2008 [29]. The first wave of BHPS comprised 5,500 private households in Britain containing 10,000 individuals. The survey was subsequently expanded to include a Northern Ireland sample from 1999, additional cases in Scotland and Wales from 2001 and a sample from the European Community Household Panel (ECHP) from 1997–2001. The BHPS followed sample members that moved within the UK to private households or institutions (excluding prisons), if they were well enough to complete an interview. Co-residents of the original sample members were also included in the survey as temporary survey members. This analysis encompasses all original and temporary survey members from the BHPS’ original sample, the additional national samples and the ECHP sample 1997–2000 (excluding ECHP Northern Ireland members). It includes only respondents aged 18+ years who participated at two adjacent waves of the survey.

### Residential mobility

BHPS respondents that changed residential address, one or more times, during the one year period between adjacent BHPS waves were categorised as ‘movers’; ‘stayers’ were people that didn’t move between waves. Mobility was defined using a derived ‘individual mover status’ variable and, when data were missing, time at current address.

To increase the numbers of movers in the dataset, data were pooled from ten subsets of adjacent waves of the survey from 1996–2006 (Waves 6–16) [14, 16, 30]. This time period was selected to coincide with that of the neighbourhood deprivation measure selected for analysis (2001).

### Difficult life event mover types

‘Difficult life event movers’ were participants that experienced one, or more, difficult life event during the time period of their move. Three types of difficult life events,—relationship breakdown, housing eviction or repossession, and job loss—were identified. Moves were defined as related to relationship breakdown if movers stated a ‘split from partner’ was the reason for their move or they had changed partnership status between waves indicating that they had divorced/separated or were no longer ‘living in a couple’. Moves following housing eviction or repossession were identified from respondents’ stated reasons for moving. Moves were identified as related to job loss if respondents’ employment status changed between waves from ‘employed’/‘self-employed’ to ‘unemployed’. Movers that had not experienced a difficult life event were categorised as ‘other movers’.

### Deprivation mover types

Deprivation mover types categorised movers dependent on whether the neighbourhood they moved to had more, less or similar levels of socio-economic deprivation than their neighbourhood of origin. Neighbourhood deprivation was defined using the Carstairs 2001 index score of the Census Area Statistics (CAS) wards of residence (n = 10,654; approximate mean 2001 population = 6,000). Carstairs score, based on four measures from the Census (male unemployment, overcrowded households, no car households and low social class), was used to categorise CAS wards into deprivation quintiles. Moves were defined as to neighbourhoods with more, less, or the same level of deprivation based upon Carstairs quintile of residence at the waves preceding and following moves.

### Mental health status

Mental health status was defined using a self-assessed measure of mental health problems. Respondents were categorised as having a mental health problem if they stated that they had a health problem and selected “anxiety, depression or bad nerves, psychiatric problems…” from a list of health issues.

### Socio-demographic variables

The socio-demographic ‘control’ variables used in this analysis were ‘five-year’ age group (18–24, 25–29… 85–89, 90+), sex, highest academic qualification and Registrar General’s Social Class. These variables were selected because they are associated with health risk but were unlikely to change as a result of difficult life events during the time period of the move (in contrast to, for example, marital status, household type, economic activity, income and housing tenure). The other variable categories used in the models are described in Table 1. Where the socio-demographic variables were missing data from more than 1% of total observations a ‘missing’ data category was used in the analysis.

**Table 1:** Descriptive data for total sample and mover types.

Mobility, health and socio-demographic variables				Total		Stayers	Movers		
						Total	Total	Difficult life event movers	Other movers
				N (obs.)	%	%	%	%	%
**Total**				122,892	100.0	-	-	-	-
**Stayers**	**Total**			111,918	91.5	100.0	-	-	-
**Movers**	**Total**			10,974	8.5	-	100.0	-	-
	**Difficult life event mover type**	**Difficult life event movers**	**Total** ^**a**^	1,630	1.2	-	14.1	100.0	-
			**Moves associated with relationship breakdown**	875	0.6	-	7.6	53.4	-
			**Moves associated with eviction and repossession**	616	0.4	-	5.3	37.3	-
			**Moves associated with job loss**	206	0.2	-	1.8	12.8	-
		**Other movers**	**Total**	9,344	7.3	-	85.9	-	100.0
	**Deprivation mover type**	**Movers within Carstairs quintile**		5,150	3.9	-	45.3	49.9	44.6
		**Movers to more deprived Carstairs quintile**		2,718	2.2	-	26.3	27.8	26.0
		**Movers to less deprived Carstairs quintile**		3,106	2.4	-	28.4	22.3	29.4
**Mental health problems**	**Yes**			10140	7.7	7.6	8.5	12.8	7.7
	**No**			112752	92.3	92.4	91.6	87.2	92.3
**Age group in years**	**18–29**			23207	16.4	13.8	44.8	44.3	44.9
	**30–44**			37478	28.8	28.6	31.0	34.1	30.5
	**45–59**			30921	25.7	26.8	13.5	16.2	13.1
	**60+**			31286	29.1	30.8	10.7	5.4	11.6
**Sex**	**Female**			66861	54.3	54.5	52.6	51.8	52.8
	**Male**			56031	45.7	45.5	47.4	48.3	47.2
**Highest academic qualification**	**Degree or diploma**			23298	18.8	18.3	24.4	20.4	25.1
	**'A-level' or equivalent**			21621	16.9	16.2	24.6	23.7	24.8
	**'O-Level', CSE or equivalent**			34672	28.9	28.8	30.3	34.4	29.6
	**None of these**			39185	32.0	33.4	17.7	18.2	17.6
	**Missing**			4116	3.3	3.4	3.0	3.3	2.9
**Registrar General’s Social Class**	**Professional**			5185	4.3	4.3	4.8	4.7	4.8
	**Managerial and technical**			33149	28.0	28.0	28.4	23.4	29.2
	**Skilled non-manual**			28163	23.7	23.7	24.6	24.2	24.6
	**Skilled manual**			22622	18.0	18.2	16.4	17.3	16.3
	**Partly skilled**			20047	15.7	15.7	16.4	19.3	15.9
	**Unskilled**			7460	5.8	5.9	4.5	5.9	4.3
	**Missing or other**			2977	1.9	1.9	2.2	2.6	2.1
	**Never worked**			3289	2.5	2.4	2.8	2.5	2.8

^a^Numbers of relationship breakdown, eviction and repossession and job loss movers do not sum to the total for difficult life event movers because a small minority of difficult life event movers experienced more than one type of difficult life event.

### Analysis strategy

Binary logistic regression models were first used to compare different types of movers. Models assessed the risk of mental health problems (dependent variable) among difficult life event movers relative to other movers. Models next assessed the odds that difficult life event movers (dependent variable) relocated to more or less deprived areas relative to moves to similarly deprived areas. The last model compared all mover types and stayers. This model assessed the odds of poor mental health (dependent variable) among difficult life event and deprivation mover types relative to stayers. All models were adjusted for socio-demographic variables. The health and socio-demographic variables in the descriptive data and models were measured at the survey wave preceding the move. Finally, to test whether poor health related to difficult life events was sustained among movers time trends in mental health problems were considered. Percentages of mental health problems were compared between mover types and stayers at the three survey waves preceding and following mobility. This analysis included only movers and stayers that were respondents at six consecutive waves of the survey.

Among the pooled dataset from ten adjacent BHPS waves there were a total of 138,109 BHPS respondents at the wave preceding moves. Among this group 12,193 (8.8%) were excluded because they did not participate at the next wave, following moves, due to non-eligibility, failure to trace, non-response or death. Among those participating at adjacent waves 3,024 were excluded because of missing data (for mental health problems (N = 891), age (N = 5), weights (N = 1,627), CAS ward at the wave preceding moves (N = 184) and following moves (N = 200) or because of inconsistent data for CAS ward of residence and mover status (N = 396)), retaining 122,892 cases in the analysis. These total observations were based upon data from 21,346 unique individuals. The analysis of time trends in health included only the 85,353 cases for which there were survey responses across six consecutive survey waves.

All percentages and odds presented in the analysis are weighted by BHPS cross-sectional weights for the wave preceding the move. These weights account for the survey sample design and non-response attrition [29]. All analysis was completed in Stata 12.1.

The Institute of Social and Economic Research, University of Essex, administers the collection, storage and use of BHPS data in accordance with the Ethical Guidelines of the Social Research Association and is obliged to conform to UK legislation regarding the handling and use of personal data. The BHPS data used in this analysis were anonymised and linked geographical identifiers were provided subject to conditions to ensure confidentiality.

## Results

### How frequent are moves related to difficult life events and moves to more and less deprived neighbourhoods?

Over the one year time period between waves of the BHPS, 8.5% of total observations moved residence (Table 1). Difficult life event movers comprised 1.2% of total cases and 14.1% of total movers. The most common type of difficult life event associated with moves was relationship breakdown, followed by eviction or repossession and job loss, affecting 53.4%, 37.3% and 12.8% of total difficult life event movers respectively. Moves associated with eviction were 3.7 times more common than those associated with repossession (data not shown). People moving within Carstairs quintiles, to more deprived quintiles and to less deprived quintiles comprised 3.9%, 2.2% and 2.4% of total observations and 45.3%, 26.3% and 28.4% of total movers respectively.

### Do people whose moves are associated with difficult life events have worse mental health than other movers?

Difficult life event movers were more likely to have poor mental health than other mover types with 12.8% reporting mental health problems compared to 7.7% of other movers and 7.6% of stayers (Table 1). Socio-demographically adjusted binary logistic regression models assessing odds of poor health among movers (Table 2) demonstrate that the odds of mental health problems among movers experiencing difficult life events were significantly elevated at 1.67 (95% CI 1.35–2.07) relative to other movers.

**Table 2:** Logistic regression odds ratios for mental health problems by difficult life event mover type.

Predicting poor health^a^	Mental health problems (yes)		
	OR	95% CI	
		lower	upper
**Difficult life event mover type**			
**Other movers**	1		
**Difficult life event movers**	1.67	1.35	2.07

^a^Adjusted for five year age group, sex, education and social class

### Are people whose moves are associated with difficult life events more likely than other movers to relocate to more deprived areas and less likely to move to less deprived areas?

Difficult life event movers compared to other movers were more likely to move to more deprived areas (27.8% versus 26.0%) or similarly deprived areas (49.9% versus 44.6%) but less likely to move to less deprived areas (22.3% versus 29.4%) (Table 1). Adjusted odds ratios indicate that difficult life event movers compared to other movers were not significantly more likely to move to more deprived areas than to similarly deprived areas but had significantly lower odds of moving to less deprived areas of 0.70 (95% CI 0.58–0.84) (Table 3).

**Table 3:** Logistic regression odds ratios for difficult live event mover by Carstairs move type.

Predicting mover type^a^	Difficult life event mover type (difficult life event movers)		
	OR	95% CI	
		lower	upper
**Deprivation mover type**			
**Movers within quintile**	1		
**Movers to more deprived quintile**	1.01	0.84	1.20
**Movers to less deprived quintile**	0.70	0.58	0.84

^a^Adjusted for five year age group, sex, education and social class

### Do people who move to more deprived areas following difficult life events have worse mental health than other mover types?

Adjusted odds indicated that mental health problems were significantly elevated among all mover groups relative to stayers, except other movers moving to less deprived areas (Table 4). The odds of poor mental health were more elevated among difficult life event movers than other movers regardless of whether they were moving to more, less or similarly deprived areas. The highest odds of mental health problems were among difficult life event movers moving to more deprived areas, followed by those moving to less and similarly deprived areas at 2.40 (95% CI 1.63–3.53), 2.17 (95% CI 1.47–3.20) and 2.02 (95% CI 1.55–2.64) respectively.

**Table 4:** Logistic regression odds ratios for mental health problems by difficult life event and Carstairs move type.

Predicting poor health^a^		Mental health problems (yes)		
		OR	95% CI	
			lower	upper
**Stayers**		1		
**Mover within quintile**	**Difficult life event movers**	2.02	1.55	2.64
	**Other movers**	1.25	1.08	1.46
**Mover to more deprived quintile**	**Difficult life event movers**	2.40	1.63	3.53
	**Other movers**	1.50	1.23	1.83
**Mover to less deprived quintile**	**Difficult life event movers**	2.17	1.47	3.20
	**Other movers**	1.10	0.93	1.29

^a^Adjusted for five year age group, sex, education and social class

### Does the relationship between poor mental health and moves following difficult life events change over time?

**Fig 1:**
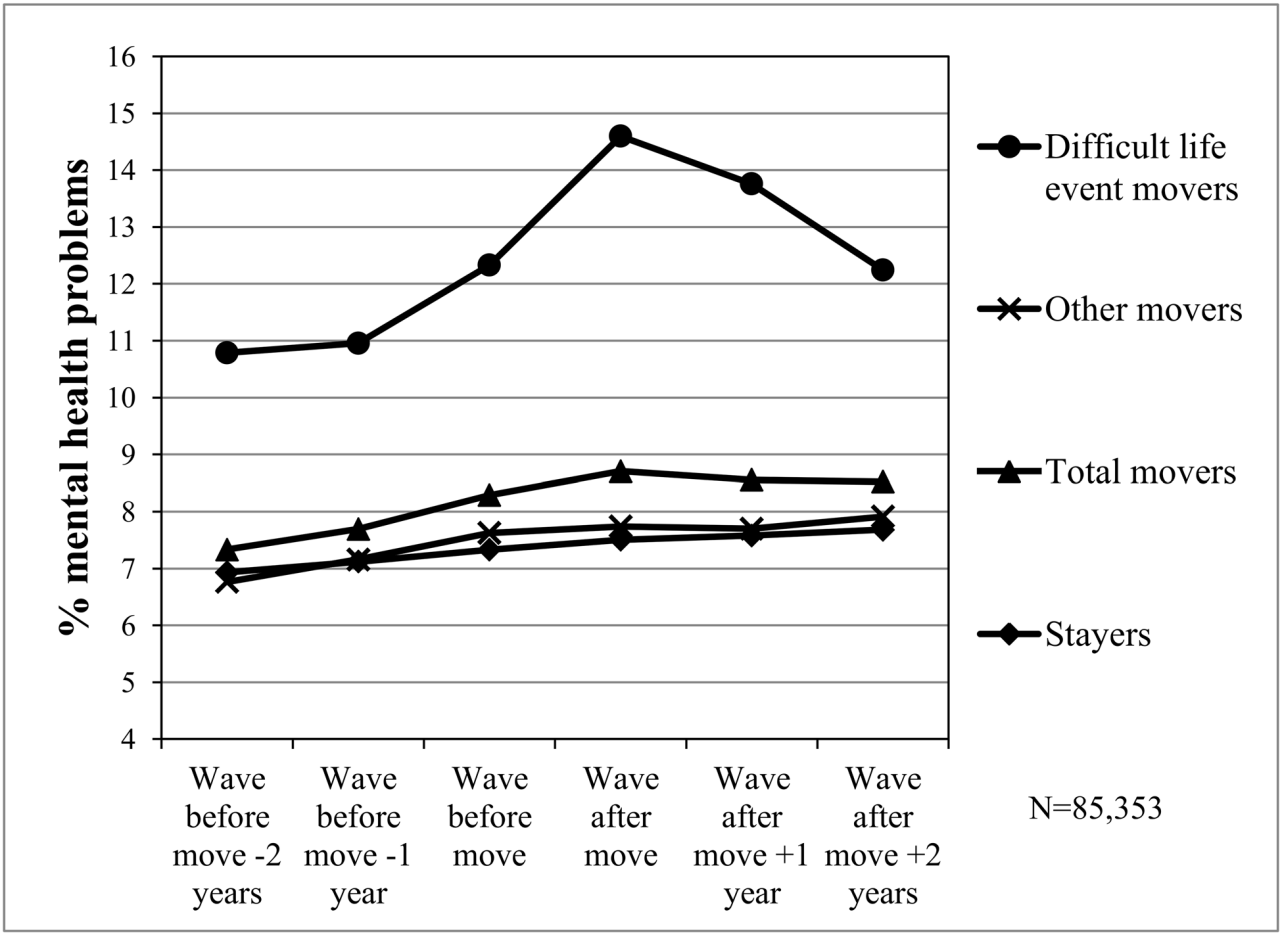
Percentage mental health problems among difficult life event mover types preceding and following moves. Mover types.

Among difficult life event movers there was a large rise in the proportion with mental health problems in the year preceding moves and the year in which moves took place (Fig 1). During this two year time period the percentage of difficult life event movers with mental health problems rose from 11.0% to 14.6%. In the two years following moves the percentage of difficult life event movers with mental health problems then fell to 12.2%. Among other movers there was little change in the proportion with mental health problems before, during and after moves.

## Discussion

This study demonstrates that relationship breakdown, housing eviction and repossession and job loss are associated with risk of mental health problems among movers and distinctive patterns of socio-spatial mobility between neighbourhoods within the UK. The analysis finds that people that moved when experiencing difficult life events were more likely to have mental health problems than other movers. Difficult life event movers were not more likely to move to a more socio-economically deprived neighbourhood but were less likely to move to an area with lower levels of deprivation than other movers. Risk of poor mental health was elevated among difficult life event movers relocating to more, less and similarly deprived areas but was most elevated among those moving to more deprived areas. Longitudinal analysis of the risk of mental health problems suggests that the poor mental health of difficult life event movers rose during the time period of the event and then subsequently fell but was still moderately elevated two years following mobility.

Previous research has found an association between residential mobility and poor mental health [5, 6, 28, 31]. It has been suggested that poor health among movers may reflect the stress of relocation itself [31] and, among people with severe mental illness, may be related to hospital admissions [32]. This study demonstrates that another reason movers may have high rates of mental health problems is that some types of difficult life events both trigger residential moves and damage mental health.

Research into selective migration and individual health has commonly focussed upon understanding how movers’ socio-demographic and health characteristics prior to relocation determine propensity to move [8]. Similarly, analysis of the impacts of migration upon inequalities in health between areas has often considered migrants’ characteristics only at one time period and assessed how mobility sorts individuals with ‘set’ characteristics into different types of neighbourhoods [8]. However, this study suggests that movers undergoing relationship breakdown, housing and job loss are likely to be experiencing significant changes in both their mental health and socio-demographic status, before, during and after moves. For these people their mobility is an intimate part of events that may form a ‘critical period’ in their life course that alters their life trajectory and has important longer term implications for their health [33].

Notably, this research found that the main effect of difficult life events upon mover’s socio-spatial trajectories was a reduced likelihood of moving to a less deprived neighbourhood. This suggests that these life events may effect individual health by constraining ‘up-ward’ trajectories and reducing the chance of residence in better environments. When considering the factors that shape selective patterns of migration further attention should be paid to events that perpetuate the ‘selective entrapment’ of residents in poor environments [34].

Previous research has indicated that stressful life events contribute to differences in mental health found between individuals with different socioeconomic status [35]. This analysis suggests that difficult life events may also contribute to the processes through which *areas* with different levels of deprivation accumulate health advantage and disadvantage. Research that has considered the association between mental health of area populations and neighbourhood deprivation has focussed primarily upon assessing the contextual effects of neighbourhood environments upon residents’ health [36–42]. However, this analysis indicates that the impacts of difficult life events upon patterns of residential mobility may result in greater concentrations of people with mental health problems in more socio-economically deprived neighbourhoods. It provides further evidence that we should consider the ‘reciprocal associations’ [43] between individual and area health. In particular, analysis assessing neighbourhood effects on health may misrepresent neighbourhood-health relationships if the factors which shape selective residential mobility are neglected [44].

Results from this analysis are consistent with the hypothesis that difficult life events trigger patterns of residential mobility that help to generate inequalities in rates of mental health found between more and less socio-economically deprived neighbourhoods. However, selective patterns of migration between neighbourhoods with different levels of disadvantage do not necessary alter geographical inequalities because migrants in- and out- of areas may be similar in number and characteristics [2, 45]. Previous analysis of BHPS data has assessed the impacts of moves over a one year time period upon inequalities in mental health problems and general health between neighbourhoods in the UK with different levels of environmental disadvantage [46]. This analysis found that residential mobility in the short term had little effect on health inequalities between the most and least advantaged areas.

Further research would be needed to assess how moves following difficult life events impact upon spatial inequalities in mental health. Results from the current analysis suggest however that the role of difficult life events in shaping area health inequalities will be limited by the small proportion of moves which are related to these events. Our results are consistent with previous research that has found the majority of moves in the UK are linked to positive life factors, including marriage, cohabitation, child birth, education and employment, and improved life satisfaction [16, 30, 47]. Moves towards more deprived areas may not be harmful for individual health if they are for beneficial reasons and support a positive life course trajectory. Some movers, particularly young adults, may choose to live in deprived neighbourhoods as better quality housing is more affordable in these areas or because they offer educational and employment opportunities that may support their subsequent well-being [48–50].

This study is one of the first to assess the link between mental health, reasons for moves and area socio-economic inequalities. It benefits from detailed longitudinal data from the BHPS, but also has limitations. The analysis considers only one measure of mental health based upon self-report. The BHPS, in common with other longitudinal surveys, has greater attrition among more mobile and unhealthy respondents [29, 51]. The cross-sectional weights used in this analysis would not have fully compensated for this bias. Residents of Northern Ireland were also under-represented in the sample. Some stated ‘splits from partner’ may not have happened between the adjacent survey waves when moves took place. In most of the analysis mental health problems were only considered at the wave preceding difficult life events and moves. The analysis also contained only a small number of socio-demographic variables measured again at one time point prior to moves. Residual socio-demographic confounding is likely to explain in part the high risk of poor mental health found among difficult life event movers and people moving to more, and similarly, deprived areas.

The analysis combines three types of difficult life events and a range of age groups that may have varied relationships with health and residential mobility. Results from this analysis may not be generalizable to all types of difficult life events or age groups. Further analysis could build upon these results to expand the evidence regarding the impacts of reasons for moves upon selective patterns of migration by considering a broader range of positive and negative reasons for moving, stratified by age group.

## Conclusions

This study indicates that difficult life events may influence the health characteristics of migrants and their socio-spatial trajectories, reducing the likelihood of moves to less deprived areas among people with mental illness. Difficult life events could contribute, modestly, to inequalities in rates of mental health problems between more and less socio-economically deprived neighbourhoods within the UK. Further assessment of the reasons that moves take place is likely to support understanding of the health selective migration processes that influence inequalities in health between areas.
